# An Unusual Case of Isolated Pancreatitis Secondary to Blunt Abdominal Trauma

**DOI:** 10.7759/cureus.23717

**Published:** 2022-03-31

**Authors:** Haroutiun Hamzoian, Reuben Plasencia, Ndifreke Ekpa, Aswin Srinivasan, Georg Elias

**Affiliations:** 1 Internal Medicine, University of Houston / HCA Healthcare Kingwood, Houston, USA; 2 Gastroenterology, University of Houston / HCA Healthcare Kingwood, Houston, USA

**Keywords:** penetrating trauma, pancreatic tail, torso trauma, acute pancreatitis, intra-abdominal injury, idiopathic pancreatitis, traumatic pancreatitis, pancreatic tail injury, blunt abdominal trauma, pancreatitis

## Abstract

Acute pancreatitis is an inflammatory condition affecting a large population and resulting in one of the most common causes of gastrointestinal hospitalizations in the United States. The pathogenesis resulting in pancreatic injury has multiple etiologies with gallstones and alcohol consumption contributing to a large majority of cases. Consequently, one uncommon cause of acute pancreatitis, direct abdominal trauma, often gets overlooked. This case describes a 20-year-old male with no past medical history or surgical interventions presenting to the hospital with two days worth of abdominal pain. Physical exam was negative for erythema, ecchymosis and lacerations but further questioning revealed a recent history of an altercation resulting in multiple blunt blows to the abdomen. CT abdomen with contrast was positive for an edematous appearing pancreatic tail with surrounding soft tissue stranding; no pancreatic fluid collections, normal gallbladder and no intrahepatic or extrahepatic biliary ductal dilation. Pancreatitis has a multitude of etiologies and practitioners should address the insulting event as well as the pathological sequelae to prevent reoccurrence of the condition. The importance of taking a full and thorough history should not be overlooked; this could lead to misdiagnosis and misjudgment of the underlying pathological process. We propose the notion that there is a significant number of patients diagnosed with idiopathic pancreatitis who may have more accurately been diagnosed with traumatic pancreatitis with a more thorough history. Additionally, due to the pancreas’s retroperitoneal location, isolated injury with abdominal trauma, such as in this patient, is quite rare. It is critical to differentiate the underlying cause of acute pancreatitis to further counsel patients about avoidance of precipitating factors. We would like to stress the importance of obtaining a thorough history and ruling out alternative causes of patient presentation as management differs greatly after treatment of the acute phase reaction. In patients with traumatic pancreatitis, physicians should establish that there were no residual abdominal injuries and advise patients to restrain from any activities that would result in any sequential abdominal trauma.

## Introduction

Acute pancreatitis is an inflammatory condition affecting a large population, resulting in one of the most common causes of gastrointestinal hospitalizations in the United States. The pathogenesis resulting in pancreatic injury has multiple etiologies with gallstones and alcohol consumption contributing to a large majority of cases. Consequently, one uncommon cause of acute pancreatitis, direct abdominal trauma, often gets overlooked [1]. This case describes a 20-year-old male presenting to the hospital with isolated pancreatic tail injury after multiple blows to his abdomen.

## Case presentation

A 20-year-old male with no past medical history or surgical interventions presented to the hospital with two days worth of abdominal pain. He ranked his pain at a 5/10 and stated that it was located in the epigastric area radiating to the left side of his abdomen. There were no relieving or exacerbating factors. The patient denied smoking, alcohol and illicit drug usage. On presentation to the emergency department, he was tachycardic, afebrile, normotensive with a pulse oximeter saturation greater than 92% on room air. No fever, diarrhea, chest pain, shortness of breath, hematemesis or hematochezia were noted on review of systems. Physical examination was negative for erythema, ecchymosis and lacerations. He did not take any medication and had no allergies. During admission to the hospital, the patient was questioned about events prior to the presentation of his symptoms. The young man stated that approximately 36 hours prior to his hospital visit, he got into an altercation with another individual and was involved in a bare-knuckle fistfight that resulted in him getting punched in the abdomen multiple times. His pain started 24 hours after the altercation and did not resolve leading to his subsequent hospitalization. Complete metabolic panel on presentation was within normal limits with a calcium level of 8.8 mg/dL. Complete blood count (CBC) revealed a slightly elevated white blood cell (WBC) count of 12,600 per microliter of blood but was otherwise unremarkable. Lipase was 3576 U/L, coronavirus disease 2019 (COVID-19) RNA testing was negative, rapid troponin was negative and lipid profile revealed a triglyceride level of 90 mg/dL, cholesterol of 129 mg/dL, high-density lipoprotein (HDL) of 24 mg/dL and low-density lipoprotein (LDL) of 73.69 mg/dL. Abdominal ultrasound was negative for gallstones, pericholecystic fluid, biliary ductal enlargement, hydronephrosis and nephrolithiasis. Imaging revealed a normally distended gallbladder [5.34 x 2.39 x 2.79cm], 2mm gallbladder wall size and 4mm common bile duct size; all measurements were within normal limits. CT abdomen with contrast was positive for an edematous appearing pancreatic tail with surrounding soft tissue stranding; no pancreatic fluid collections, normal gallbladder and no intrahepatic or extrahepatic biliary ductal dilation. Patient was made NPO, started on Lactated Ringer's solution 150mls/hr and started on pain control with acetaminophen 650 mg every 6h and ibuprofen 600 mg every 6h. After 24 hours of treatment, the patient’s pain had subsided to a 2/10. His tachycardia had resolved and a repeat CBC revealed a WBC count that was within normal limits. He was counseled on injury prevention to prevent future pancreatitis sequelae. 

## Discussion

The patient was examined and lab work was reviewed for many known causes of acute pancreatitis. The patient was questioned about ethanol, steroid, prescription and illicit drug usage which he denied. This healthy 20-year-old male did not have any past medical history, open abdominal procedures, endoscopic retrograde cholangiopancreatographies or laparoscopic interventions. Hypercalcemia was ruled out with a complete metabolic panel and hypertriglyceridemia was ruled out with a normal lipid panel. Furthermore, abdominal ultrasound did not show any evidence of gallstones or obstructive sequelae. Physical inspection did not show any erythema, hyperemia or hematomas present on the external abdominal wall. Inspection was negative for Grey-Turner’s and Cullenʼs signs. The only notable finding was the history of blunt abdominal trauma a day prior to the presentation of symptoms which was acquired through a thorough and detailed history. It is important to note that gallstones and alcohol abuse account for 70% of cases of acute pancreatitis [1]. Hypertriglyceridemia accounts for 2-5% and a large percentage of cases fall under the umbrella term idiopathic. With this case, we would like to stress the importance of taking a thorough history and not simply treating the presenting condition; this could lead to misdiagnosis and misjudgment of the underlying pathological process. Pancreatitis has a multitude of etiologies and practitioners should address the insulting event as well as the pathological sequelae to prevent reoccurrence of the condition. We propose the notion that there is a significant number of patients diagnosed with idiopathic pancreatitis who may have more accurately been diagnosed with traumatic pancreatitis with a more thorough history. 

**Figure 1:**
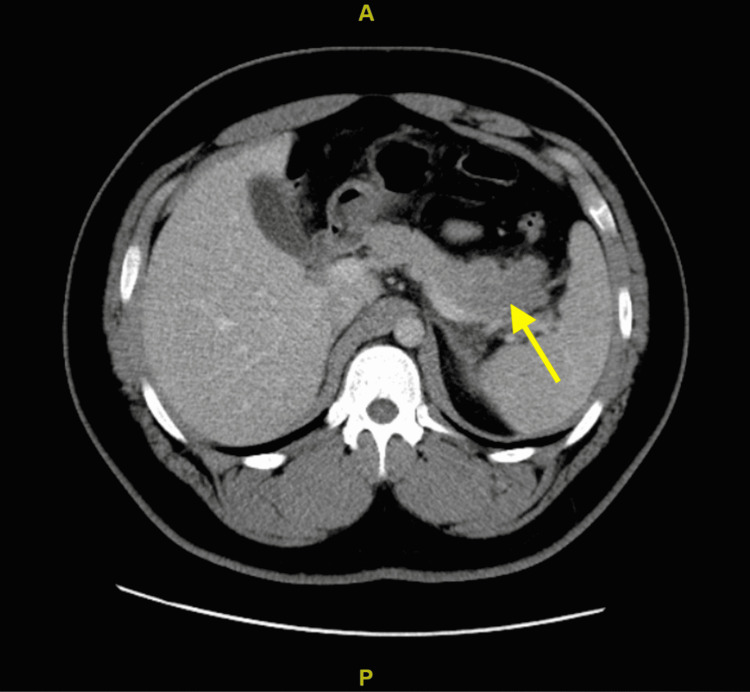
Pancreatic Tail Injury on CT Imaging

In addition, we would like to stress the specifics of this patient’s pancreatitis as literature review did not reveal many cases with similar manifestations. It is important to note that pancreatic trauma occurs in only 4% of all patients sustaining abdominal injuries. Patients usually present with vague symptoms and injury associated with other abdominal viscera. Due to the pancreas’s retroperitoneal location, isolated injury with abdominal trauma is quite rare [2]. Pancreatic injury is usually found with a combination of hepatic (47% of cases), gastric (42%), major vascular (41%), splenic (28%), renal (23%), and duodenal (19%) anatomic injury [3,4]. It is also important to note the specific type of trauma and the correlated anatomical manifestations seen on the pancreas. A study published in the *Scandinavian Journal of Surgery *found that 61.3% of cases of traumatic pancreatitis were secondary to blunt trauma and 38.7% of cases due to penetrating trauma. When compared to blunt trauma, penetrating trauma was more commonly found to involve the pancreatic tail [5]. CT imaging of our patient revealed an edematous appearing pancreatic tail with surrounding soft tissue stranding; no pancreatic fluid collections, normal gallbladder and no intrahepatic or extrahepatic biliary ductal injury (Figure 1). Subsequently, this case of isolated pancreatic tail injury is a rare presentation of acute pancreatitis especially in a patient who suffered blunt abdominal trauma and no penetrating injury. Bedside Index for Severity in Acute Pancreatitis (BISAP) score was calculated and found to be 0, estimating a <1% mortality [6]. Patient was clinically improving and his pain was subsiding with the administration of IV fluids and pain control. On the day of discharge, he was educated on pancreatitis, its causes and possible complications. He was instructed to restrain from activity that could exacerbate his symptoms and counseled on avoidance of future trauma to the abdominal wall.

## Conclusions

This case stresses the importance of differentiating the underlying cause of acute pancreatitis to further counsel patients about avoidance of precipitating factors. We would like to stress the importance of obtaining a thorough history and ruling out alternative causes of patient presentation as management differs greatly after treatment of the acute phase reaction. In patients with traumatic pancreatitis, physicians should establish that there were no residual abdominal injuries and advise patients to restrain from any activities that would result in any sequential abdominal trauma.
